# Cloaking the ACE2 receptor with salivary cationic proteins inhibits SARS-CoV-2 entry

**DOI:** 10.1093/jb/mvac054

**Published:** 2022-07-06

**Authors:** Katsutoshi Yoshizato, Toshio Taira, Misako Sato-Matsubara, Shizuko Sekiguchi, Yoriko Yabunaka, Yukimi Kira, Tetsu Ohashi, Atsuko Daikoku, Ken Ofusa, Chiho Kadono, Daisuke Oikawa, Tsutomu Matsubara, Yu Nakagama, Yasutoshi Kido, Fuminori Tokunaga, Kazuo Ikeda, Akira Kaneko, Norifumi Kawada

**Affiliations:** Donated Laboratory for Synthetic Biology, Graduate School of Medicine, Osaka Metropolitan University, Osaka 545-8585, Japan; BioIntegrence Co., Graduate School of Medicine, Osaka Metropolitan University, Osaka 545-8585, Japan; Sapporo Division, Cosmo Bio Co., Ltd, Otaru, Hokkaido 047-0261, Japan; Donated Laboratory for Synthetic Biology, Graduate School of Medicine, Osaka Metropolitan University, Osaka 545-8585, Japan; Department of Hepatology, Graduate School of Medicine, Osaka Metropolitan University, Osaka 545-8585, Japan; Sapporo Division, Cosmo Bio Co., Ltd, Otaru, Hokkaido 047-0261, Japan; Department of Research Support Platform, Graduate School of Medicine, Osaka Metropolitan University, Osaka 545-8585, Japan; Department of Research Support Platform, Graduate School of Medicine, Osaka Metropolitan University, Osaka 545-8585, Japan; Sapporo Division, Cosmo Bio Co., Ltd, Otaru, Hokkaido 047-0261, Japan; Anatomy and Regenerative Biology, Graduate School of Medicine, Osaka Metropolitan University, Osaka 545-8585, Japan; Laboratory of Foods and Life Sciences, IDEA Consultants, Inc., Osaka 559-8519, Japan; Donated Laboratory for Synthetic Biology, Graduate School of Medicine, Osaka Metropolitan University, Osaka 545-8585, Japan; Department of Hepatology, Graduate School of Medicine, Osaka Metropolitan University, Osaka 545-8585, Japan; Department of Pathobiochemistry, Graduate School of Medicine, Osaka Metropolitan University, Osaka 545-8585, Japan; Anatomy and Regenerative Biology, Graduate School of Medicine, Osaka Metropolitan University, Osaka 545-8585, Japan; Department of Parasitology and Research Center for Infectious Disease Sciences, Graduate School of Medicine, Osaka Metropolitan University, Osaka 545-8585, Japan; Department of Parasitology and Research Center for Infectious Disease Sciences, Graduate School of Medicine, Osaka Metropolitan University, Osaka 545-8585, Japan; Department of Pathobiochemistry, Graduate School of Medicine, Osaka Metropolitan University, Osaka 545-8585, Japan; Anatomy and Regenerative Biology, Graduate School of Medicine, Osaka Metropolitan University, Osaka 545-8585, Japan; Department of Parasitology and Research Center for Infectious Disease Sciences, Graduate School of Medicine, Osaka Metropolitan University, Osaka 545-8585, Japan; Department of Hepatology, Graduate School of Medicine, Osaka Metropolitan University, Osaka 545-8585, Japan

**Keywords:** SARS-CoV-2 spike protein, saliva, neutrophil elastase, histone H2A, angiotensin-converting enzyme 2

## Abstract

Saliva contributes to the innate immune system, which suggests that it can prevent SARS-CoV-2 entry. We studied the ability of healthy salivary proteins to bind to angiotensin-converting enzyme 2 (ACE2) using biolayer interferometry and pull-down assays. Their effects on binding between the receptor-binding domain of the SARS-CoV-2 spike protein S1 (S1) and ACE2 were determined using an enzyme-linked immunosorbent assay. Saliva bound to ACE2 and disrupted the binding of S1 to ACE2 and four ACE2-binding salivary proteins were identified, including cationic histone H2A and neutrophil elastase, which inhibited the S1-ACE2 interaction. Calf thymus histone (ct-histone) also inhibited binding as effectively as histone H2A. The results of a cell-based infection assay indicated that ct-histone suppressed SARS-CoV-2 pseudoviral invasion into ACE2-expressing host cells. Manufactured polypeptides, such as ε-poly-L-lysine, also disrupted S1-ACE2 binding, indicating the importance of the cationic properties of salivary proteins in ACE2 binding. Overall, we demonstrated that positively charged salivary proteins are a barrier against SARS-CoV-2 entry by cloaking the negatively charged surface of ACE2 and provided a view that the cationic polypeptides represent a preventative and therapeutic treatment against COVID-19.

## Graphical Abstract

**Graphical Abstract:**
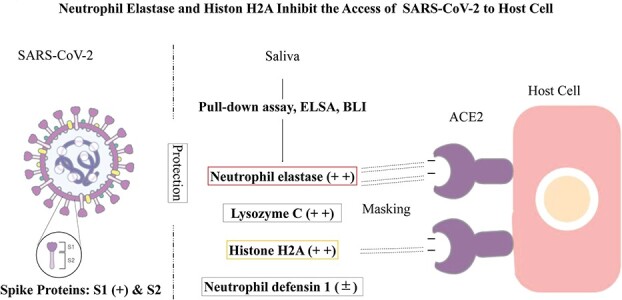

## Abbreviations

ACE2: angiotensin-converting enzyme 2
BLI: biolayer interferometry
CBB stain: Coomassie brilliant blue stain
COVID-19: coronavirus disease 2019
ct-histone: calf thymus histone
DMEM: Dulbecco’s modified Eagle’s medium
DW: distilled water
ELISA: enzyme-linked immunosorbent assay
FPLC: first protein liquid chromatography
GuHCl: guanidinium hydrochloride
IC_50_: half maximal (50%) inhibitory concentration
KD: dissociation constant
PBS: phosphate buffered saline
pI: isoelectric point
MS/MS: tandem mass spectrometry
MW: molecular weight
RBD: receptor-binding domain
rh: recombinant human
RT: room temperature
S1: spike S1 glycoprotein
SARS-CoV-2: severe acute respiratory syndrome coronavirus 2
SD: standard deviation
SDS-PAGE: polyacrylamide gel electrophoresis in the presence of sodium dodecyl sulphate
SE: standard error
TFA: trifluoroacetic acid
TOF-MS: time of flight mass spectrometer
Tris: tris(hydroxymethyl)aminomethane
UF: ultrafiltration
TFA: trifluoreacetic acid
ACN: acetonitrile

##  

The human body contains multiple defence mechanisms to combat invading pathogens (*1*). Saliva contains various defence-related proteins including immunoglobulins, lysozymes and defensins and functions as a frontline defence mechanism against invasive pathogens (*2*). The outbreak of coronavirus disease 2019 (COVID-19), which is caused by severe acute respiratory syndrome coronavirus 2 (SARS-CoV-2) infection, has threatened human life worldwide. Determining the mechanism by which SARS-CoV-2 escapes the multiple barriers of the innate defence system is necessary to decrease the infection rate of this virus. Studies have shown that children and younger adults are less susceptible than the elderly and are often asymptomatic or show mild symptoms following infection (*3*, *4*). The mortality rate is higher in the older population (*5*). These age-dependent tendencies are not well understood (*6*–*8*).

The quantity and quality of saliva change over time (*9*). Unstimulated normal saliva flow rates are significantly lower in elderly adults compared with younger adults (*10*). Saliva from children contains more innate immune-related substances with antibacterial/antiviral activity compared with that from elderly individuals (*11*, *12*). Therefore, we hypothesized that saliva contains molecules that protect against SARS-CoV-2 entry into host cells.

Viral infection begins with the binding of the receptor-binding domain (RBD) of the SARS-CoV-2 spike S1 glycoprotein (S1) to angiotensin-converting enzyme 2 (ACE2) expressed on host cell membranes (*13*). Previous studies have indicated that binding occurs via the salt bridge between the positively charged RBD and negatively charged site of ACE (*14*–*17*). In the present study, we show that saliva hinders the access of S1 to ACE2 and identified four ACE2-binding salivary proteins, of which cationic histone H2A and neutrophil elastase inhibited S1-ACE2 binding.

We hypothesize that salivary cationic proteins act as a frontline innate immune barrier against SARS-CoV-2 entry by cloaking the ACE2 receptor. This study can establish a basis for the further development of manufactured cationic polypeptides as preventatives and treatments for COVID-19.

## Materials and Methods

### Proteins, peptides, chemicals and reagents

Recombinant (r) SARS-CoV-2 spike Fc-fused S1 (wild type) and recombinant human (rh)-ACE2 with a His-Tag were purchased from Sino Biological, Inc. (Wayne, PA, USA). S1 and ACE2 consist of Arg319-Phe541 (RBD, NCBI Reference Sequence: YP_009724390.1) and Met1-Ser740 (NP_068576.1 including SAR-CoV-2 spike glycoprotein interactive regions), respectively. The following materials were obtained from Sigma-Aldrich (Saint Louis, MO, USA): rh-Histone H2A, neutrophil elastase (EC3.4.21.37), BSA, poly-L-arginine HCl (MW > 70 kDa) and calf thymus histone [(ct-histone) histone type IIS, a heterogenous mixture of all the histone families (H1, H2A, H2B, H3, H4)]. rh-Lysozyme was from FUJIFILM Wako Pure Chemicals Co. (Osaka, Japan). Synthetic h-α-defensin-1 and elastatinal, a neutrophil elastase inhibitor (IC_50_ for elastase = 1.25 μM), were purchased from the Peptide Institute (Osaka, Japan). Sequence-grade trypsin was from Promega (Madison, WI). α-Poly-L-lysine (16 lysine residues long, 2.6 kDa), ε-poly-L-lysine (a homo-polymeric compound composed of 30 lysine monomers linked at ε-amino groups, 4.5 kDa) and L-arginine octapeptides (MW = ~1.4 kDa) were supplied by Cosmo Bio Co., Ltd (Tokyo, Japan). Polybrene was purchased from NACARAI TESQUE (Kyoto, Japan). Kits for silver staining (2D-Silver Stain Reagent II and CBB staining) [Page Blue 83 Stain Reagent (CBB-R250)] were from Cosmo Bio Co., Ltd. BCA protein assay kits were from Thermo Fisher Scientific (Tokyo, Japan). All other chemicals not specified were from FUJIFILM Wako Pure Chemicals Co. The amino acid sequences and pIs were determined using the UniProtKB and Compute pI/Mw tools from Expasy, respectively.

### Collection and treatment of saliva and tears

Saliva and tear samples were collected with approval from the ethics committee of Osaka Metropolitan University Graduate School of Medicine (approval number #2020-003) and the ethics committee of Cosmo Bio Co., Ltd (approval numbers #20210201 and #20181207, respectively). Samples were collected from SARS-CoV-2-negative adult donors who provided written informed consent to participate. Saliva was collected from eight healthy donors with the following age distribution and sex: two females aged 20–39 years, two females, one male aged 40–60 years, two males aged 61 years and one male aged 77 years. Tear samples were collected from two healthy male and female donors >40 years of age. Saliva was collected in test tubes as drool before a meal from the mouth without any taste stimulation. The saliva samples were centrifuged at 6700 × g for 15 min and the supernatants were used for analysis or stored at −20°C until use. Approximately 100 μl of shed tears were collected into injection tubes and used for analysis or stored at −20°C until use. The protein concentrations were determined using a BCA protein assay kit (Thermo Fisher Scientific).

### ELISA of S1-ACE2 binding

Ni-NTA-coated, transparent 96-well plates (Qiagen, Tokyo, Japan) were used to immobilize rhACE2. ACE2 was dissolved at 1 μg/ml in PBS containing 3% BSA and 0.02% Tween 20 (assay buffer) and added to the Ni plate wells (50 μl/well). The plates were incubated at room temperature (RT) for 2 h and washed three times with 200 μl/well PBS containing 0.02% Tween 20 (wash buffer). To measure S1-binding to ACE2, various amounts of Fc-fused r-S1 were dissolved in assay buffer and added to the ACE2-immobilized wells at 50 μl/well and incubated at RT for 1 h. The wells were washed three times with 200 μl of wash buffer, then 50 μl of assay buffer containing HRP-conjugated Protein A (diluted 4000-fold, Proteintech Group, Inc.) were added and incubated at RT for 2 h. The wells were then washed three times. The bound S1 was measured with a plate reader (Tecan Infinite 200Pro) at 450 nm. The effects of saliva on S1-ACE2 binding were quantified as follows. The ACE2-immobilized wells were pretreated with saliva, which had been appropriately diluted with PBS containing 3% BSA and 0.02% Tween 20, followed by incubation with 100 μl of 100 ng/ml S1 in PBS for 60 min at RT. The OD_450_ of the wells were measured as OD_test_. The bound S1 in the absence of the test samples was also measured as OD_test = 0_. S1-ACE2 inhibition curves were generated so that the ordinate and abscissa indicated the % maximum binding (calculated by dividing the OD_test_ by the OD_test = 0_) and the log_10_ of the protein concentrations (μg/ml) of the test samples, respectively. Where necessary, the IC_50_ (μg/ml) was calculated using GraphPad Prism 9.1.0 with sigmoidal four-parameter logistic (4PL).

### BLI assays for ACE2 binding capacity

All BLI experiments were performed using Ni-NTA biosensors (Biosensors/Ni-NTA, Sartorius) with an OctetRED96 system. The assay materials were prepared in PBS containing 0.01% BSA and 0.002% Tween 20. Assays were performed at 30°C and shaking at 1000 rpm. His-tagged ACE2 was immobilized on Ni-NTA sensors. To determine the effect of saliva on S1 and ACE2 binding, ACE2 immobilized sensors were pretreated with various concentrations of saliva (Donor 7) and then exposed to 5 nM of S1. Binding kinetic constants for ACE2 against S1or Histone H2A were determined at various concentrations of analytes. The effects of histone H2A were also examined. The data were analysed by ForteBio Analysis Software (version 10.0). To create the final binding curves, signals for the reference samples and biosensors were subtracted from the raw binding signals. Each dissociation constant (KD) value was calculated by a 1:1 fitting model on global analysis.

### Fractionation of saliva by ultrafiltration for analysis by ELISA and HPLC

Saliva is highly viscous and glutinous and not easy to handle biochemically. Therefore, we first treated it with guanidinium hydrochloride (GuHCl), according to Takehara *et al.* (*18*), followed by fractionation using ultrafiltration (UF) and an Amicon Ultra-filter. For small-scale experiments, 1.2 ml of saliva from Donor 1 was mixed with 2.4 ml of GT buffer (6 M GuHCl in 10-mM Tris buffer, pH 6.5), incubated overnight at 4°C, placed on a 100 kDa filter and centrifuged at 14,000 × g for 20 min at 4°C. The GT buffer of the retentate was replaced with 6 M urea-10 mM Tris buffer, pH 6.5 (UT-buffer), followed by PBS using repeated centrifugation through the filter. The retentate in 400 μl PBS was used as Fraction I (Frac-I, 100 kDa < fraction). The 100 kDa flow-through fraction was subjected to UF as above using a 10 kDa filter and its retentate in 400-μl PBS was used as Frac-II (10 kDa < fraction < 100 kDa). The flow-through fraction of 10 kDa UF was dialyzed against distilled water (DW), lyophilized, dissolved in 400-μl PBS and used as Frac-III (10 kDa > fraction). The protein concentrations of these three UF fractions were determined using a BCA protein assay (Thermo Fisher Scientific). Proteins in the saliva, Frac-I, II and III, each 11 μg, were separated on 1% SDS-PAGE gels with a gradient ranging from 4% to 20% on precast gels [Cosmo Bio Co., Ltd, MULTIGEL II mini 4/20 (13 W)]. The gels were stained with silver stain.

The large-scale fractionation was carried out to obtain larger amounts of Frac-II and III using 4 ml of saliva. Saliva was mixed with 8 ml of GT buffer. The fractionation by UF was performed as above, except that the device was centrifuged at 5000 × g for 60 min. The 100 kDa-flow-through fraction was placed on a 10 kDa filter and centrifuged, and the retentate was recovered in 2 ml of UT as Frac-II. The 10 kDa-flow-through fraction was pooled, dialyzed against DW, lyophilized and dissolved in 500 μl of UT-buffer as Frac-III. The proteins of Frac-II and III were quantified, separated by SDS-PAGE and stained with CBB. The Frac-II in UT was subjected to reversed-phase chromatography.

### Separation of saliva proteins with S1-ACE2 binding inhibitory activity by reversed-phase chromatography

A portion (0.9 ml) of Frac-II in UT-buffer prepared from the saliva of Donor 1 was loaded onto a column (4.6 mm in diameter × 250 mm in height) of reversed-phase resin (C18) equipped with an FPLC apparatus (GE AKTA pure 25) and separated by a gradient elution buffer consisting of 0.1% trifluoroacetic acid (TFA) and acetonitrile (ACN) at a flow rate of 1.0 ml/min. The absorbance was measured at 280 nm (protein concentrations). The eluate was collected in tubes (1.0 ml/tube) and dried in a centrifugal evaporator (Eyela UT1000, Tokyo Rikakikai Co. Ltd, Tokyo, Japan) followed by dissolving in 50 μl of UT-buffer. The fractions were diluted 25-fold with PBS containing 3% BSA and 0.02% Tween 20 (the urea concentration was reduced to 240 mM) and used for ELISA to determine S1-ACE2 binding. The other fractions of the eluate in UT-buffer were diluted 2-fold in 2× SDS-PAGE sample buffer and used for SDS-PAGE analysis.

### Isolation of ACE2-binding salivary proteins by pull-down assay

Fresh saliva (4 ml) from Donors 7 and 8 was mixed with 8 ml of 6 M GuHCl overnight at 4°C, dialyzed three times against 300 ml of 6 M urea at 4°C for 3 h and centrifuged at 5000 × g for 30 min. Half of the supernatants in 6 M urea [SUP in urea] were dialyzed against 1 L of PBS at 4°C overnight and the resulting dialysate, [GuHCl-treated saliva], was used as ‘saliva’. The other half of SUP in urea was centrifuged at 6000 × g for 40 min in a Vivaspin 20 UF unit with a 100 kDa filter (Sartorius Stedim Lab Ltd, UK). The obtained flow-through fraction was dialyzed at overnight at 4°C against 1 l of PBS and the resulting dialysate was designated the ‘100 kDa > fraction’ (100 > F).

ACE2 was immobilized on Ni-NTA magnetic agarose beads as follows. The beads (1 mg, His 60 Ni magnetic beads; Takara, Japan) were incubated at 4°C for 3 h in 500 μl of phosphate buffer (50-mM NaH_2_PO_4_/2H_2_O, 300 mM NaCl, pH 8.0) containing rhACE2 (250 ng/ml). The beads were washed three times with the same volume of assay buffer (0.02% Tween 20 in PBS) and 500 μl of the dialysate (protein concentrations, 100 μg/ml) was mixed with the bait on the beads and incubated for 16 h at 4°C. The beads were collected with magnets, gently washed with 1 ml of assay buffer, and the ACE2-bound proteins were eluted using 10 μl of 100-mM imidazole. The eluates were subjected to 1% SDS-PAGE at 80 V for 90 min on 4%–20% gradient gels. The gels were stained with silver stain (Silver Stain II Kit). Densitometry of the protein bands was performed using ImageJ software. Known concentrations of BSA were used to generate a standard curve.

### TOF-MS for amino acid sequencing

Proteins were separated by SDS-PAGE with silver staining after reversed-phase chromatography or pull-down assay. Selected bands were digested with trypsin and amino acid sequence analysis was done using TOF-MS as previously described (*19*, *20*). Briefly, the target protein bands were excised from the gels, rehydrated, de-stained, rinsed twice with Milli-Q water and dehydrated again with 100% ACN. The pieces were dried in a vacuum centrifuge, rehydrated in solution containing 0.1 μg/μl modified sequence-grade trypsin and incubated for 16 h at 37°C. The digestion was terminated with the addition of 10 μl of 5% TFA. Peptides were extracted, dried in a vacuum centrifuge, resuspended in 0.1% TFA and eluted from ZipTip using 50% ACN and 0.1% TFA. The eluants were spotted onto a target plate, which were mixed with a matrix solution containing 0.3 mg/ml α-cyano-hydroxycinnamic acid, 33% acetone, 66% ethanol and then completely air-dried at RT. MS and MS/MS spectra were obtained using an Ultraflex TOF/TOF mass spectrometer (Bruker Daltonics, Yokohama, Japan). An external peptide mixture was used to calibrate the instrument. Proteins were identified using MASCOT software (Matrix Science) and the NCBInr database.

### Assays for SARS-CoV-2 S pseudovirus invasion into host cells

SARS-CoV-2 S pseudoviruses were prepared as follows: Lenti-X 293 cells (Takara Bio USA, Inc.) were transfected using Lenti-X SARS-CoV-2 packaging single shots (D614G Spike, Truncated, Takara Bio USA, Inc.) either with the pLVXS-ZsGreen-puro or pLVXS-Luciferase-puro vector, according to the manufacturer’s instructions. Briefly, pseudovirus supernatants were collected approximately 48 h post-transfection, centrifuged and filtered through a 0.45-μm low protein-binding filter (Minisart, Non-pyrogenic, S7598-FXOSK, Sartourius Stedim, Gottingen, Germany). Pseudoviruses were concentrated using a Lenti-X concentrator (Takara Bio USA, Inc.) in PBS to obtain a 50-fold concentrated virus density. They were then divided into aliquots and stored at −80°C until use.

HEK293T cells stably expressing ACE2 fused with tdTomato on its C terminus were prepared and used as host cells (293-ACE2-tdTomato-expressing cells) for a pseudoviral invasion assay. HEK293T cells were cultured in DMEM containing 10% FBS were co-transfected with the expression vector, pcDNA3-ACE2-tdTomato and a puromycin-resistant vector, pXS-Puro. After 48 h, the cells were treated with puromycin (1 mg/ml) and cells were selected by limiting dilution and propagated. The expression of ACE2-tdTomato was confirmed using immunoblotting and fluorescence imaging, which indicates that most cells were tdTomato-positive.

The 293-ACE2-tdTomato-expressing cells were treated with varied concentrations of ct-histone (up to 400 μg/ml) for 12 h at 37°C, washed with PBS and incubated for 48 h in culture medium containing the viral solution and polybrene (6 μg/ml). Pseudoviruses within cells were observed using a ZsGreen1 fluorescence filter with an IX70 inverted Olympus microscope and luciferase activity was quantitated using the One-Glo luciferase assay system (Promega). The quantity of the invaded pseudoviruses was expressed as relative luminescence units and normalized to the expression level of tdTomato, which was measured at 554/581 and presented as RLU/Fluo.

### Statistical analysis

For quantitation experiments, the measurements were done in triplicate and the results are shown as the average with SD. For graphic representation, the error bars indicate the SD. IC_50_ values from the ELISAs were determined from sigmoidal inhibition curves by 4PL models using GraphPad Prism Software ver. 9.1.0. A significance test was performed using a two-way ANOVA, indicated as ^∗∗^*P* < 0.01 and ^∗∗∗^*P* < 0.001.

## Results

### Inhibition of S1-ACE2 binding by saliva and tears

**Fig. 1:**
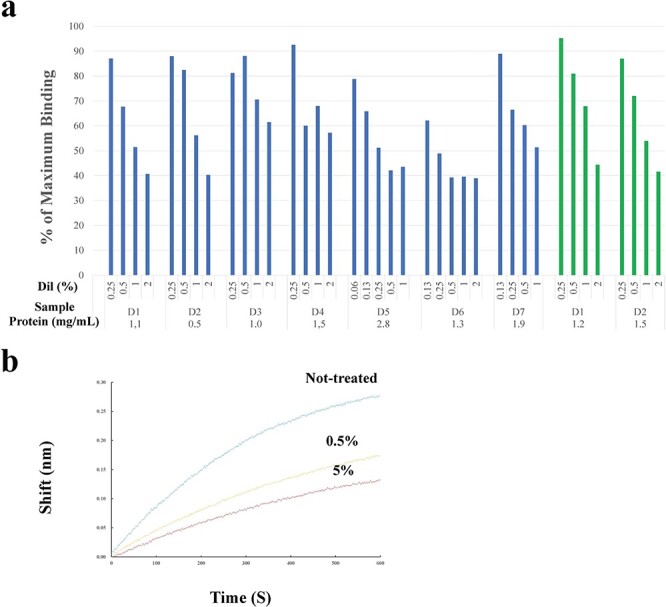
**Effects of saliva and tears on S1-ACE2 binding.** a. ELISA. Saliva (blue bars) and tears (green bars) were diluted with PBS and incubated with ACE2 immobilized in wells, which were then incubated with S1. Bound S1 was optically quantified. The extent of S1 binding (vertical axis) was expressed as the % maximum binding (the ratio of OD in the presence of saliva/tears against that in its absence). ‘D’ represents the donor who provided the saliva or tears. The numerals shown under each bar indicate the dilution rate [Dil (%)] (0.25%–2%) of each test sample and those at the bottom of the abscissa represent the concentrations (μg/ml) of the original (non-diluted) samples. Saliva was tested for 11 donors. Data from seven cases are depicted here. Tears were tested for two donors. b. BLI assay. ACE2-immobilized sensors were pretreated with diluted saliva from done #7: the blue, yellow and red lines represent non-treated, diluted to 0.5% and 5%, respectively. The concentration of the original saliva was 3.2 mg/ml. The sensors were then incubated with 5 nM of S1 and the amount of S1 bound to the sensors were determined. The BLI assay was performed twice, which yielded similar results and one of these is presented here.

Preliminary studies have demonstrated the effects of various kinds of bodily fluids, such as milk (bovines and humans), sera (bovines and humans), tears (cats and humans) and saliva (humans), on S1-ACE2 binding using ELISA, in which immobilized ACE2 was pretreated with each fluids and ACE2-bound S1 was quantitated. Of these, tears (both cat and human) and saliva suppressed the interaction. Therefore, saliva and tears were collected from several donors and systematically examined for their effects on S1-ACE2 binding (Fig. 1a). All of the tested saliva and tears inhibited binding in a dose-dependent manner. Inhibition of S1-ACE2 binding by saliva was also demonstrated in a BLI assay (Fig. 1b). Saliva was selected as a source to further investigate the biochemical nature responsible for the inhibition of S1-ACE2 binding because it is readily available in sufficient amounts for experiments.

### Inhibition of S1-ACE2 binding by UF-separated salivary fractions

**Fig. 2:**
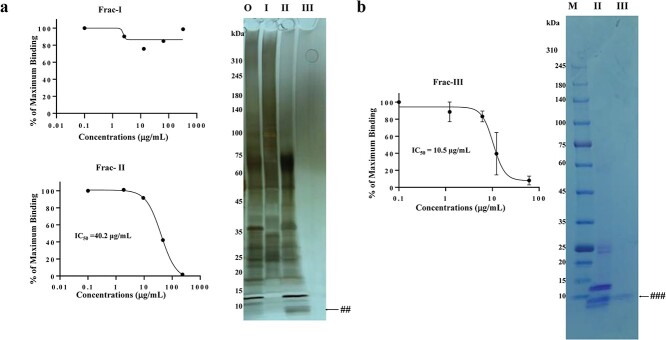
**Inhibition of S1-ACE2 binding by UF-fractionated fractions.** a. Frac-I, II and III were prepared from donor #1 saliva on a small scale were subjected to ELISA at the indicated concentrations. The ELISA was conducted three times. No inhibition was observed for Frac-I and typical inhibition curves were obtained for Frac-II. The calculated IC_50_ value of Frac-II was 40.2 μg/ml. The proteins of these fractions together with original (O, unfractionated) were separated by SDS-PAGE by loading 1-μg protein for O, I, II and III and stained by silver (right panel). ## indicates the band corresponding to the band indicated by the blue arrowhead in Fig. 3b. M, MW markers. b. Frac-II and III were prepared from Donor #1 saliva at a larger scale. ELISA experiments (left panel) showed that Frac-III exhibits a sigmoidal inhibition curve with an IC_50_ value of 10.5 μg/ml. The right panel showed CBB-stained SDS-PAGE of these Frac-II (II) and III (III), in which portions of 10 μl containing 558 μg/ml protein (Frac-II) and 58.9 μg/ml of protein (Frac-III) were analysed. ### indicates the band corresponding to the band indicated by ## in a II and the blue arrowhead in Fig. 3b. M, MW markers.

Saliva (Donor 1) was separated into three fractions by UF in a small scale, resulting in Frac-I (100 kDa < fraction), Frac-II (10 kDa < fraction < 100 kDa) and Frac-III (10 kDa > fraction). The recovery of the original salivary proteins in Frac-I, II and III was 28%, 11% and 2%, respectively. ELISA revealed that Frac-I did not show binding inhibition at concentrations up to 200 μg/ml (Fig. 2a, Frac-I). In contrast, Frac-II significantly inhibited binding in a dose-dependent manner up to 200 μg/ml with an IC_50_ value of 40.2 μg/ml (Fig. 2a, Frac-II). The concentration of Frac-III prepared in this small-scale experiment was insufficient for an ELISA assay. There were no discernible distinct bands for Frac-III (Fig. 2a right panel, III). Therefore, Frac-III was similarly prepared from the same donor on a larger scale (Fig. 2b). Frac-III inhibited the binding with and IC_50_ value of 10.5 μg/ml (Fig. 2b, left panel, Frac-III). Proteins in the obtained Frac-II and III were separated by SDS-PAGE followed by CBB staining (Fig. 2b, right panel). A single band of <~10 kDa indicated by ### was observed in Frac-III as only one visible band in this fraction, which corresponded to the band marked by ## in Frac-II (Fig. 2a, right panel).

### Separation of salivary proteins by reversed-phase chromatography and pull-down assay

**Fig. 3:**
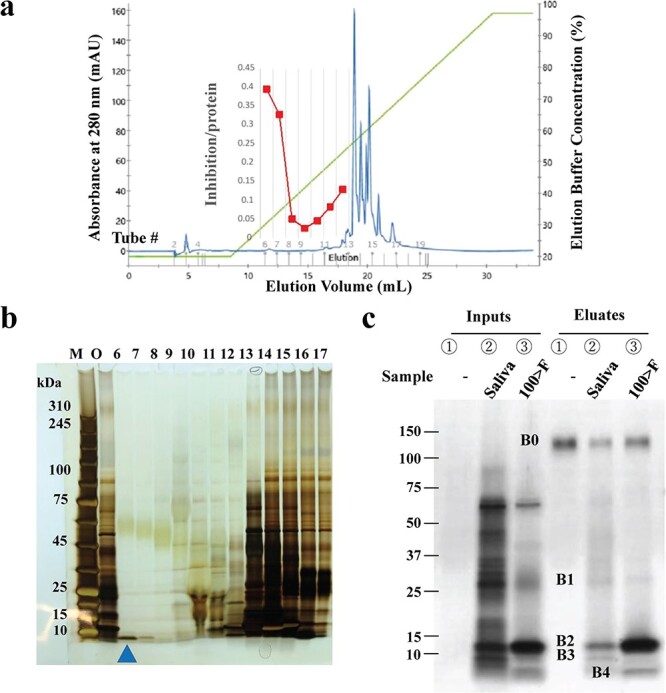
**Separation of salivary proteins by reversed-phase chromatography and pull-down assay.** a. Reversed-phase chromatography. Frac-II from Donor 1 was loaded onto the resin and eluted with a salt gradient, which was monitored by OD_280nm_ for protein concentration (blue line) and OD_210nm_ for salt concentration (green line). A total of 1 ml of each eluate was collected into tubes, portions of which were used for the ELISA to evaluate their ability to inhibit S1-ACE2 binding (red line). b. Portions of the eluates in Tubes #6 through #17 in a and indicated at the top of the lane were used for SDS-PAGE followed by silver staining. Lane M: MW marker, O: original proteins. The band with the blue arrowhead in the lane representing Tube #6 was excised for TOF-MS analysis. c. Capture of ACE2-binding salivary proteins by pull-down assay. The saliva of Donor 7 and its 100 kDa > fraction were subjected to a pull-down assay. Proteins (1 μg in 10 μl PBS/lane) were loaded as follows: Lane ①, ② and ③ in ‘Inputs’ and ‘Eluates’ represent ‘without saliva (−)’, ‘Saliva’, and ‘100 kDa > fraction (100 > F)’, respectively. The band (B0) at ~150 kDa was His-ACE2. A similar banding profile was obtained from Donor 8 saliva. The bands marked B1, B2, B3 and B4 at the left side of the gel were excised for TOF-MS. The experiments were conducted three times.

To identify a specific protein(s) that inhibits the S1-ACE2 interaction, Frac-II was separated on C18 resin (Fig. 3a). Most (~99%) of the loaded proteins were absorbed onto the resin. The eluates up to Tube #15 were then used in an ELISA to measure the effects on S1-ACE2 binding and for SDS-PAGE analysis. A high inhibitory activity was detected near the beginning of the elution (red line in Fig. 3a), in which proteins were hardly eluted (blue line in Fig. 3a); however, the activity decreased up to Tube #10, followed by a slight increase to Tube #12. Dose-dependent inhibition was not observed in Tubes #13 to #15, most likely because the loaded saliva proteins were abundantly eluted in these tubes (the blue line of Fig. 3a). Portions of the eluates from Tubes #6 through #17 were analysed by SDS-PAGE (Fig. 3b), which revealed the presence of a single band at <~10 kDa in Tube #6, as indicated by the blue arrowhead. Careful analysis of the protein separation profile (blue line in Fig. 3a) revealed a minute protein peak in Tube #6, which exerted the highest S1-ACE2 inhibition activity. The band with ### in Fig. 2b III and that with blue arrowhead in Fig. 3b seems to be derived from the same protein and this protein was considered to be entirely responsible for the inhibition. This blue arrowheaded band was excised for TOF-MS analysis and yielded a single peptide ‘AGLQFPVGR’ with a significance threshold of *P* < 0.05 (Supplementary Fig. S1a). This sequence was a complete match to the human histone H2A Type 1 sequence. Considering its MW (~6 kDa) and the nominal MW of histone H2A (14 kDa), we concluded that the band represents a degradation product of histone H2A.

The identification of histone H2A in saliva supported our hypothesis that saliva contains a protein(s) that interacts with ACE2. A pull-down assay was performed to capture ACE2-binding proteins in saliva (Donor 7). Input samples of saliva and its UF-fraction < 100 kDa (100 > F), including Frac-II and III, both had inhibitory actions on S1-ACE2 binding. The samples were incubated with ACE2-immobilized agarose and the ACE2-bound proteins were separated by SDS-PAGE (Fig. 3c). The pull-down proteins were visualized and three bands (band B1 at ~ 30 kDa, B2 at ~ 15 kDa and B3 at ~ 10 kDa) were observed. Those from the 100 kDa > fraction (100 > F) exhibited two bands: one band corresponded to B2 above, although it was much more concentrated, whereas the other (B4) migrated at ~6 kDa. Band B4 had a similar MW to the band from Tube #6, indicated by the blue arrowhead in the SDS-PAGE (Fig. 3b) and was considered histone H2A. TOF-MS analysis of B4 yielded a single peptide sequence of AGLQFPVGR (Supplementary Fig. S1b), the same sequence obtained for the band in Tube #6 (Fig. 3b). We concluded that this protein was histone H2A. Consequently, the single band with ### in Frac-III in Fig. 2b is most likely histone H2A.

TOF-MS of Bands B1 through B3 produced the sequences: QVFAVQR, SNVCTLVR and VVLGAHNLSR; AWVAWR, WESGYNTR and STDYGIFQINSR; and IPACIAGER, YGTCIYQGR and RYGTCIYQGR, respectively (Supplementary Fig. S1c–e). These sequences corresponded to human neutrophil elastase preproprotein, lysozyme C precursor and neutrophil defensin 1 preproprotein, respectively. The proteins were present in saliva in an intact form (not degraded) because their estimated MWs were similar to their nominal MWs. Using the densities of B1 through B4, the approximate concentration (μg/band) of neutrophil elastase, lysozyme C, neutrophil defensin 1 and histone H2A in saliva were estimated to be 4.5, 25, 3.5 and 1, respectively.

### Neutrophil elastase and histone H2A inhibit S1-ACE2 binding

**Fig. 4:**
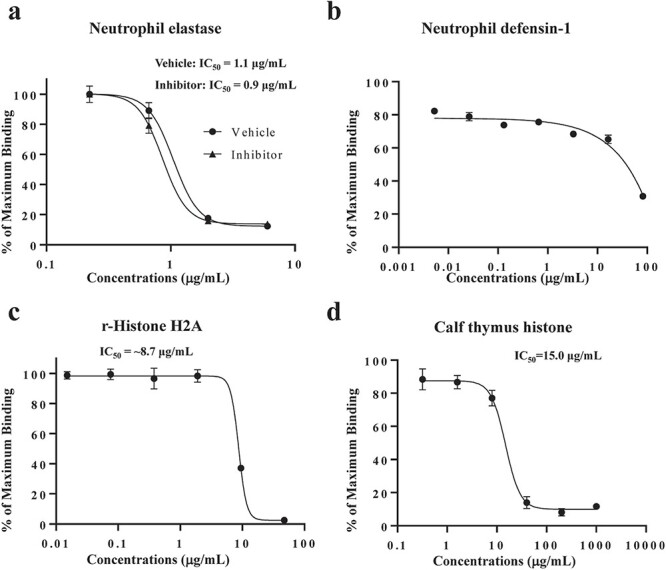
**Effects of pull-down captured proteins on S1-ACE2 binding.** ELISA was performed to determine the effects of human neutrophil elastase (a), neutrophil defensin-1 (b) and r-histone H2A (c) at the indicated concentrations. In neutrophil elastase experiments, the effects were examined in the presence (Inhibitor, 100 μM) and absence (Vehicle) of its inhibitor, elastatinal. The rate of inhibition was the average ± SD (error bar) of three determinations. The experiment was conducted twice. d. ct-histone was also inhibited S1-ACE2 binding.

ELISA was performed for the ACE2-binding proteins captured in the pull-down assay. Neutrophil elastase of h-leucocytes exhibited typical inhibitory activity in the concentration range 0.08–6.67 μg/ml with an IC_50_ value of 1.1 μg/ml (Fig. 4a). This activity appeared to be entirely independent of its enzyme activity as the presence of sufficient amounts (100 μM) of elastatinal (a specific inhibitor of neutrophil elastase) did not ameliorate the inhibition activity (Fig. 4a). r-Lysozyme C did not exhibit any activity up to 2 mg/ml (data not shown). Synthetic neutrophil defensin-1 yielded a dull inhibition curve with ~40% inhibition at 66.8 μg/ml (Fig. 4b). r-Histone H2A suppressed S1-ACE2 binding beyond ~10 μg/ml, with an IC_50_ value of ~8.7 μg/ml (Fig. 4c). In addition, native calf thymus (ct)-histone, a heterogeneous mixture of histone species (H2A, H2B, H3 and H4), inhibited binding with an IC_50_ value of 15.0 μg/ml (Fig. 4d), which was similarly effective as r-histone H2A. This indicates that the effect is not histone-subtype specific and not human-type specific.

**Fig. 5:**
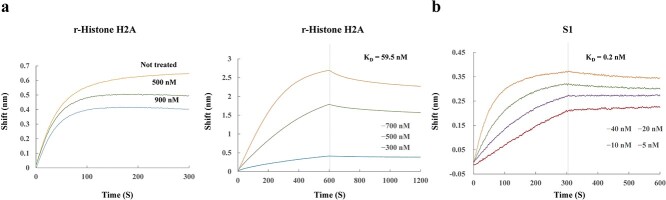
**BLI assay for the binding of r-histone H2A to ACE2.** The binding of r-histone H2A to ACE2 was also evaluated by BLI. a. Association analysis of r-histone H2A with ACE2. ACE2 sensors were incubated with r-histone H2A at 0 (orange), 500 nM (green) and 900 nM (blue). b. Kinetic analysis. ACE2-immobilized sensors were incubated with r-histone H2A at the indicated concentrations: 300 nM (blue), 500 nM (green) and 700 nM (orange) and its association and dissociation to the sensor were monitored (right panel). The KD value was calculated as 59.5 nM. Similarly, the sensors were incubated with S1: 5 nM (red), 10 nM (purple), 20 nM (green) and 40 nM (orange). The KD value was 0.2 nM. Vertical broken lines indicate the time point when the dissociation phase started. Each point represents the average of triplicate determinations with error bars (SD).

An association analysis by BLI confirmed the dose-dependent inhibition of histone H2A for S1-ACE2 binding (Fig. 5a). A subsequent kinetic analysis revealed that the dissociation constant (K_D_) of r-histone H2A against ACE2 was ~60 nM (Fig. 5b, left). A parallel BLI analysis on S1 indicated a K_D_ value of 0.2 nM (Fig. 5b, right).

### Cationic polypeptides inhibit S1-ACE2 binding

**Fig. 6:**
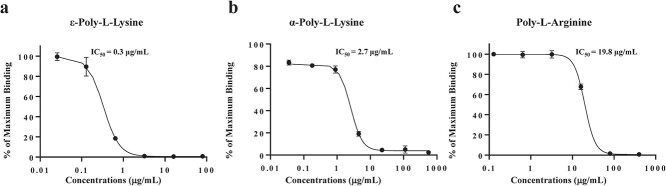
**Inhibition of S1-ACE2 binding by Lys- and Arg-based cationic polypeptides.** The effects of ε-poly-L-lysine, α- poly-L-lysine and α-poly-L-arginine on S1-ACE2 binding were examined by ELISA. Immobilized ACE2 was treated with the polypeptides at the indicated concentrations, followed by incubation with S1 (100 ng/ml) and subjected to colour development. a. ε-Poly-L-lysine: IC_50_ = 0.3 μg/ml (0.07 μM). b. α-Poly-L-lysine: IC_50_ = 2.7 μg/ml (1.02 μM). c. Poly-L-arginine: IC_50_ = 19.8 μg/ml (0.28 μM). All experiments were conducted three times at each concentration and the bars indicate the SD.

Neutrophil elastase and histone H2A are both extremely cationic, suggesting the possibility that Lys- and/or Arg-rich cationic proteins/polypeptides preferentially interact with ACE2 through intermolecular salt bridges. We tested this possibility using two types of manufactured cationic polypeptides: Lys-based cationic peptides (ε- and α-poly-L-lysine) and Arg-based peptides (α-L-arginine octapeptides and α-poly-L-arginine). ε-Poly-L-lysine robustly inhibited binding with an IC_50_ value of 0.3 μg/ml (Fig. 6a). α-Poly-L-lysine (Fig. 6b) and poly-L-arginine peptides (Fig. 6c) also inhibited binding with IC_50_ values of 2.7 and 19.8 μg/ml, respectively. However, L-arginine octapeptides did not show any such activity at concentrations up to 1 mg/ml (data not shown).

### Inhibition of SARS-CoV-2 S pseudovirus invasion into ACE2-expressing cells by histone

**Fig. 7:**
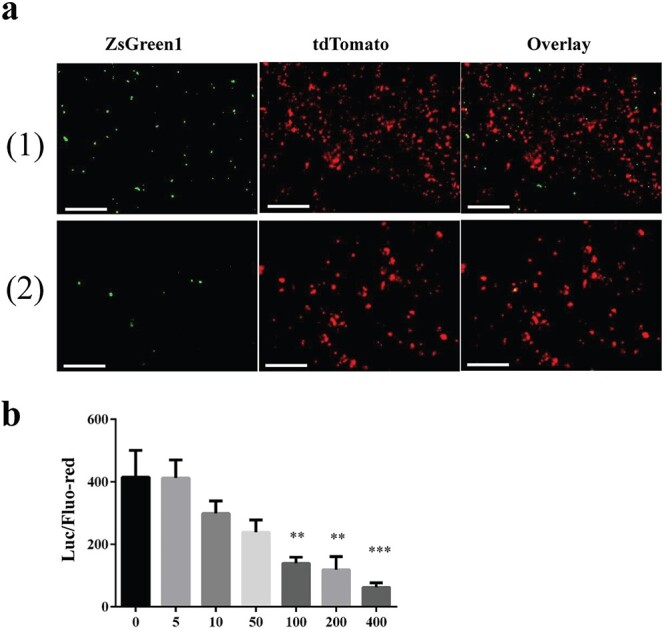
**Histone disrupts SAR-CoV2 pseudoviral entry into host cells.** ACE2/tdTomato-expressing HEK293T cells were treated with ct-histone and incubated with pseudoviruses bearing SAR-CoV2 S proteins. Invasion of pseudovirus into the cells was examined. a. Fluorescent images. The cells were incubated in the absence (*1*) or presence of ct-histone at a concentration of 360 μg/ml (*2*). Left, GFP (ZsGreen pseudovirus) images. Middle, red (293-ACE2/tdTomato cells) images. Right, overlay of left and middle. Scale bar, 100 μm. The infection experiment was performed four times and produced a similar result. A representative is presented here. b. Quantitation of pseudovirus invasion. HEK293T cells were treated with ct-histone at a range of concentrations from 0 to 400 μl/ml for 16 h. The medium was replaced with DMEM containing pseudoviruses carrying proteins with luciferase. The luciferase activity (invaded viral quantity, ‘Luc’) was normalized to the fluorescence intensity of tdTomato (quantity of ACE2-expressing cells, ‘Fluo-red’) as Luc/Fluo-red. Data were analysed using a one-way ANOVA and presented as the mean ± SD. Significant differences among groups are indicated as ^∗∗^*P* < 0.01 and ^∗∗∗^*P* < 0.001.

To determine whether the inhibition of S1-ACE2 binding by salivary cationic proteins/polypeptides occurs under ‘physiological’ conditions, we established an assay system to examine the invasion of SARS-CoV-2 S pseudoviruses into ACE2-expressing HEK293T cells and studied the effects of ct-histone on pseudovirus entry into the cells. We showed that pseudoviruses invade ACE2-expressing cells, confirming that this assay is suitable for examining viral infection within host cells (Supplementary Fig. S2). ACE2-HEK293T cells were treated with 360 μg/ml of ct-histone for 12 h and then exposed to SARS-CoV-2 S/ZsGreen1 pseudoviruses for 48 h. The virus invaded the histone-treated host cells to a lesser extent (Fig. 7a2) compared with the untreated cells (Fig. 7a1). The inhibitory activity of the histone against viral invasion was quantitated by infecting SARS-CoV-2 S/luciferase pseudoviruses into ACE2-expressing cells that were pretreated with various concentrations of histone (up to 400 μg/ml). Histone inhibited viral infection of the host cells in a dose-dependent manner. The inhibition rate reached ~80% at 400 μg/ml histone (Fig. 7b).

## Discussion

We determined that neutrophil elastase and histone H2A present in healthy adult saliva can inhibit S1-ACE2 binding. The identification of histone H2A in saliva is noteworthy. First, this protein was identified by different methods (UF-fractionation/reversed-phased chromatography and pull-down assay). Second, the inhibitory activity was demonstrated by three different assays (ELISA, BLI and pseudovirus-host cell infection test). Finally, histone H2A is highly cationic. The binding of histone H2A to ACE2 was verified by the different methods as said above, which are generally utilized for demonstrating specific interactions between different kinds of proteins.

A model for the binding mechanism of S protein with SARS-CoV from 2002 to 2003 to ACE2 (*14*) suggests that binding occurs through salt bridges that form between positively charged RBD and the negatively charged sites in the ACE2 catalytic site (*15*) (peptidase domain) (*17*), which also contains the S1-binding region (*21*). Analysis of the S protein revealed that the current SARS-CoV-2 S is more positively charged compared with that of SARS-CoV, suggesting that the former binds to the receptor with a higher affinity. This correlates well with the difference in infectivity between the two viruses (*16*). Cationic residues (Lys and Arg) in RBD and anionic residues (Glu and Asp) in the receptor are considered major contributors to salt bridge formation (*15*). This model supports our hypothesis that human neutrophil elastase and histone H2A interfere with the access of SARS-CoV-2 to ACE2-expressing host cells by masking ACE2 through salt bridge formation between these cationic salivary proteins and the negatively charged domain of ACE2.

Intestinal defensin 5 interacts with ACE2 and inhibits SARS-CoV-2 invasion (*22*). Of note, we showed that defensin 1 bound to ACE2 but hardly interfered with S1-ACE2 binding (IC_50_ > ~20 μM). The theoretical pIs of defensin 1 and 5 were 6.54 and 8.30, respectively (Table 1), indicating that defensin 5 is cationic and defensin 1 is neutral at physiological pH. We considered that the differential inhibitory activities of the two types of defensins resulted from differences in cationicity. Lysozyme C is cationic (pI = 9.38) (Table 1) and was identified as an ACE2-binding salivary protein in the present study; however, this protein did not disrupt S1-ACE2 binding. Lysozyme C was found to bind with ACE (*23*), the original member of the ACE family. Examining inability of lysozyme C to disrupt the S1-ACE2 interaction will contribute to further understanding the mechanism of S1-ACE2 binding. The fact that lysozyme C fails to inhibit S1-ACE2 binding also indicates that the effects of neutrophil elastase/histone H2A on S1-ACE2 binding is ‘specific’ in the sense that cationicity is not the sole requirement for inhibiting S1-ACE2 binding. There might be refined mechanisms in the interactions among neutrophil elastase/histone H2A, ACE2 and S1, which remains to be studied. Highly cationic ε-poly-L-lys produced by *Streptomyces albulus* showed a high rate of disruption of S1-ACE2 binding and is widely utilized as a preservation agent in food products. Our study warrants further exploration of the utilization of such industrialized cationic peptides as protective and therapeutic agents against SARS-CoV-2 infection.

**Table 1:** IC_50_ and pI of proteins/polypeptides

		**Proteins/Polypeptides**	**IC**_**50**_ **(μM)**	**SE**	**pI** ^*^	**Electronic charge** at pH = 7**
**Examined in this study**	**Salivary**	Neutrophil elastase (29 kDa***)	**0.03**	**1.08**	**9.71**	**+**
		Lysozyme C (17 kDa***)	**No inhibition**		**9.38**	**+**
		Neutrophil defensin-1 (10 kDa***)	**Faint inhibition (~20>)**		**6.54**	**+/−**
		Histone H2A type 1 (14 kDa****)	**~0.62**	**1.09**	**10.90**	**+**
	**Manufactured**	**α-Poly-L-lysine (2.6 kDa)**	**1.02**	**2.23**	**11.18**	**+**
		**ε-Poly-L-lysine (4.5 kDa)**	**0.07**	**1.06**	**~11.5**	**+**
		**α-Poly-L-arginine (>70 kDa)**	**<0.28**	**1.09**	**>13.6**	**+**
		**α-Octa-L-arginine (>1.4 kDa)**	**Faint inhibition**		**12.85**	**+**
**Related to** **this study**		Paneth cell defensin-5 (10 kDa***)			**8.30**	**+**
		**ACE peptidase domain**			**5.04**	−
		**S1 receptor binding domain**			**8.91**	**+**

^*^Theoretical values of respective proteins with the full-length.

^**^Estimated from pI.

^***^The full-length.

^****^The full-length but not degraded length (~6 kDa).

A comparison of IC_50_ values suggests that the affinity of neutrophil elastase to ACE2 is >20-fold higher compared with that of histone H2A (Table 1). Although quantitative comparison of the binding affinity of proteins/peptides to ACE2 is beyond the scope of this study, we estimated that the affinity of neutrophil elastase to ACE2 may be approximately 15-fold lower compared with that of S1 to ACE2, taking into consideration that the binding affinity of histone H2A to ACE2 measured by BLI was 300-fold lower compared with that of S1. These ‘theoretical estimations’ suggest that the affinity of neutrophil elastase and histone H2A in saliva towards ACE2 is relatively low as compared with that of S1. However, the ‘practical’ amount of these proteins in healthy saliva is likely sufficient to suppress S1-ACE2 because a substantial volume of saliva is restlessly secreted in healthy people. Therefore, salivary proteins identified as inhibitors of S1-ACE2 binding may protect ACE2 on the surface of host cells from SARS-CoV-2 spike access. The pathophysiological significance of the salivary proteins that interact with ACE2 and, as a result, suppress the access of CoV-2 S1 to the receptor, largely remains to be determined.

As an approach to explore physiological roles of salivary cationic proteins effective in inhibiting the S1-ACE2 binding, we estimated the concentration of elastase and histone H2A in healthy saliva that can bind to ACE2 from the results of pull-down assay experiments, based on the concentrations (μg/band) (4.5 and 1 for neutrophilic elastase and histone H2A, respectively) and the volume of saliva used for the assay (500 μl). This suggests that restlessly flowing saliva contains 9 and 2 μg/ml protein that could bind to ACE2, which supports our hypothesis concerning the physiological significance of these salivary proteins in protecting access of SARS-CoV-2 to host cell. An accurate determination of the physiological concentrations of the salivary proteins we discovered in this study will be the subject of a follow-up study, as well as that of their binding kinetics against ACE2.

ACE2 expressing host cells are thought to be susceptible to SARS-CoV-2 invasion because it mediates the viral entry into host cells. It has been known that ACE2 is abundant on the membrane of lung alveolar epithelial cells and enterocytes (*22*). Importantly to our study, oral cavity epithelial cells in salivary glands (cheek and tongue) and gingiva express ACE2 (*24*–*26*), indicating that the oral cavity is an important site for SARS-CoV-2 infection and saliva is a potential route of SARS-CoV-2 transmission (*24*). Therefore, we currently speculate that the cloaking effects of salivary histone H2A and neutrophil elastase we discovered in this study have pathophysiological significance in preventing the viral entry to the oral epithelial cells *in vivo*. This study can pave a way to develop salivary cationic proteins and manufactured cationic polypeptides as preventatives and treatments for COVID-19.

## Supplementary Material

Web_Material_mvac054
