# Supplementary material for: Cloaking the ACE2 receptor with salivary cationic proteins inhibits SARS-CoV-2 entry
Source: J Biochem. 2022 Jul 6;172(4):205–16. doi: 10.1093/jb/mvac054 (PMC9278198; doi:10.1093/jb/mvac054)
Supplement: Web_Material_mvac054 [file web_material_mvac054.zip › Supplementary Fig. 1 2022.6.21.10.44.upload pdf.pdf]

Supplementary Fig. 1a. Mascot Search Results for the band indicated by blue arrowhead in Fig. 3b.

Mascot Search Results

User	: 10kDa
Email	:
Search title	:
MS data file	: DATA.TXT
Database	: NCBI_RefSeq_human RefSeq_human_170130 (111280 sequences; 74376932 residues)
Timestamp	: 29 Oct 2020 at 09:27:43 GMT
Significant hits:	gi|4504239 histone H2A type 1 [Homo sapiens]

Probability Based Mowse Score

Ions score is -10*Log(P), where P is the probability that the observed match is a random event.
Individual ions scores > 37 indicate identity or extensive homology (p<0.05).
Protein scores are derived from ions scores as a non-probabilistic basis for ranking protein hits.

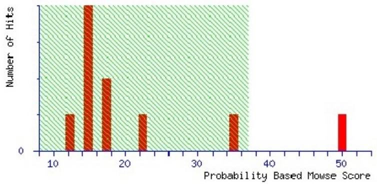

Peptide Summary Report

Switch to Protein Summary Report

To create a bookmark for this report, right click this link: Peptide Summary Report (../data/20201029/F062281.dat)

Select All | Select None | Search Selected | ☐ Error tolerant | Archive Report

1. gi|4504239 — **Mass:** 14083 **Score:** 50 **Peptides matched:** 1
histone H2A type 1 [Homo sapiens]
☐ Check to include this hit in error tolerant search or archive report

	Query	Observed	Mr(expt)	Mr(calc)	Delta	Miss	Score	Expect	Rank	Peptide
☑	23	944.53	943.52	943.52	-0.00	0	50	0.0028	1	AGLQFPVGR

Proteins matching the same set of peptides:

Accession / Description	Mass	Score	Peptides matched
gi|4504241 histone H2A type 1 [Homo sapiens]	14083	50	1
gi|4504243 histone H2A type 1 [Homo sapiens]	14083	50	1
gi|4504245 histone H2A type 1-C [Homo sapiens]	14097	50	1
gi|4504249 histone H2A type 1 [Homo sapiens]	14083	50	1
gi|4504251 histone H2A type 2-A [Homo sapiens]	14087	50	1
gi|4504253 histone H2AX [Homo sapiens]	15135	50	1
gi|4504255 histone H2A.Z [Homo sapiens]	13545	50	1
gi|6912616 histone H2A.V isoform 1 [Homo sapiens]	13501	50	1
gi|10645195 histone H2A type 1-B/E [Homo sapiens]	14127	50	1
gi|10800130 histone H2A type 1-D [Homo sapiens]	14099	50	1
gi|10800132 histone H2A type 1 [Homo sapiens]	14083	50	1
gi|10800144 histone cluster 1, H2aj [Homo sapiens]	13928	50	1
gi|15617199 histone H2A type 3 [Homo sapiens]	14113	50	1
gi|18105045 histone H2A type 1-H [Homo sapiens]	13898	50	1
gi|19557656 histone H2A type 1-B/E [Homo sapiens]	14127	50	1
gi|20357599 histone H2A.V isoform 2 [Homo sapiens]	12196	50	1
gi|24638446 histone H2A type 2-C [Homo sapiens]	13980	50	1
gi|25092737 histone H2A type 1-A [Homo sapiens]	14225	50	1
gi|28195394 histone H2A type 2-B [Homo sapiens]	13987	50	1

Supplementary Fig. 1b. Mascot Search Results for B4 in Fig. 3c

Mascot Search Results

```
User           : 10kDa1
Email          :
Search title   :
MS data file   : DATA.TXT
Database       : NCBI_RefSeq_human RefSeq_human_170130 (111280 sequences; 74376932 residues)
Timestamp      : 21 Jun 2021 at 09:17:11 GMT
Significant hits: gi|4504239  histone H2A type 1 [Homo sapiens]
```

Probability Based Mowse Score

Ions score is -10*Log(P), where P is the probability that the observed match is a random event.
Individual ions scores > 37 indicate identity or extensive homology (p<0.05).
Protein scores are derived from ions scores as a non-probabilistic basis for ranking protein hits.

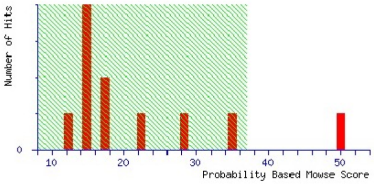

Peptide Summary Report

Switch to Protein Summary Report

To create a bookmark for this report, right click this link: Peptide Summary Report (../data/20201029/F062281.dat)

Select All | Select None | Search Selected | ☐Error tolerant | Archive Report

1. gi|4504239 — Mass: 14083 Score: 50 Peptides matched: 1
histone H2A type 1 [Homo sapiens]
☐ Check to include this hit in error tolerant search or archive report

	Query	Observed	Mr(expt)	Mr(calc)	Delta	Miss	Score	Expect	Rank	Peptide
☑	23	944.53	943.52	943.52	-0.00	0	50	0.0028	1	AGLQFPVGR

Proteins matching the same set of peptides:

Accession / Description	Mass	Score	Peptides matched
gi|4504241 histone H2A type 1 [Homo sapiens]	14083	50	1
gi|4504243 histone H2A type 1 [Homo sapiens]	14083	50	1
gi|4504245 histone H2A type 1-C [Homo sapiens]	14097	50	1
gi|4504249 histone H2A type 1 [Homo sapiens]	14083	50	1
gi|4504251 histone H2A type 2-A [Homo sapiens]	14087	50	1
gi|4504253 histone H2AX [Homo sapiens]	15135	50	1
gi|4504255 histone H2A.Z [Homo sapiens]	13545	50	1
gi|6912616 histone H2A.V isoform 1 [Homo sapiens]	13501	50	1
gi|10645195 histone H2A type 1-B/E [Homo sapiens]	14127	50	1
gi|10800130 histone H2A type 1-D [Homo sapiens]	14099	50	1
gi|10800132 histone H2A type 1 [Homo sapiens]	14083	50	1
gi|10800144 histone cluster 1, H2aj [Homo sapiens]	13928	50	1
gi|15617199 histone H2A type 3 [Homo sapiens]	14113	50	1
gi|18105045 histone H2A type 1-H [Homo sapiens]	13898	50	1
gi|19557656 histone H2A type 1-B/E [Homo sapiens]	14127	50	1
gi|20357599 histone H2A.V isoform 2 [Homo sapiens]	12196	50	1
gi|24638446 histone H2A type 2-C [Homo sapiens]	13980	50	1
gi|25092737 histone H2A type 1-A [Homo sapiens]	14225	50	1
gi|28195394 histone H2A type 2-B [Homo sapiens]	13987	50	1

Supplementary Fig. 1c. Mascot Search Results for B1 in Fig. 3c.

{MATRIX SCIENCE} Mascot Search Results

User : YZ2
Email :
Search title :
MS data file : DATA.TXT
Database : NCBI_RefSeq_human RefSeq_human_170130 (111280 sequences; 74376932 residues)
Timestamp : 10 Dec 2020 at 05:08:39 GMT
Significant hits: gi|4503549 neutrophil elastase preproprotein [Homo sapiens]

Probability Based Mowse Score

Ions score is -10*Log(P), where P is the probability that the observed match is a random event.
Individual ions scores > 36 indicate identity or extensive homology (p<0.05).
Protein scores are derived from ions scores as a non-probabilistic basis for ranking protein hits.

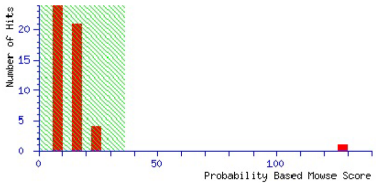

Peptide Summary Report

Switch to Protein Summary Report

To create a bookmark for this report, right click this link: Peptide Summary Report (../data/20201210/F062345.dat)

Select All | Select None | Search Selected | ☐ Error tolerant | Archive Report

1. gi|4503549 **Mass:** 29127 **Score:** 128 **Peptides matched:** 4
neutrophil elastase preproprotein [Homo sapiens]
☐ Check to include this hit in error tolerant search or archive report

	Query	Observed	Mr(expt)	Mr(calc)	Delta	Miss	Score	Expect	Rank	Peptide
☑	17	847.48	846.47	846.47	-0.00	0	38	0.05	1	QVFAVQR
☑	25	948.49	947.48	947.49	-0.00	0	29	0.34	1	SNVCTLVR
☑	32	1065.62	1064.61	1064.61	0.00	0	49	0.0015	1	VVLGAHNLSR
	35	1104.59	1103.59	1103.59	-0.00	1	11	19	4	RSNVCTLVR

Proteins matching the same set of peptides:

gi|768001224 **Mass:** 29127 **Score:** 128 **Peptides matched:** 4
PREDICTED: neutrophil elastase isoform X1 [Homo sapiens]

gi|768001228 **Mass:** 29127 **Score:** 128 **Peptides matched:** 4
PREDICTED: neutrophil elastase isoform X1 [Homo sapiens]

Peptide matches not assigned to protein hits: (no details means no match)
List sorted by Decreasing Score. Sort by Decreasing Intensity or Increasing Query # / Mr

	Query	Observed	Mr(expt)	Mr(calc)	Delta	Miss	Score	Expect	Rank	Peptide
☑	15	830.45	829.44	829.48	-0.04	0	22	2.4	1	MLLAVQR
☑	24	927.49	926.48	926.49	-0.00	0	22	1.3	1	YLYEIAR
☑	39	1542.82	1541.81	1541.77	0.04	1	17	3.4	1	DFTAAFPRMLTTR + Oxidation (M)
☑	13	749.40	748.39	748.43	-0.04	1	15	11	1	ITTMRK
☑	35	1104.59	1103.59	1103.59	-0.00	1	14	9.9	1	RMNSLTLNR
☑	69	1897.93	1896.93	1896.97	-0.04	0	13	4.7	1	EPPASPSLPQHLDLSGPR
☑	37	1246.67	1245.66	1245.67	-0.01	0	9	28	1	HPHDLVILMR + Oxidation (M)
☑	53	1797.90	1796.89	1796.88	0.01	0	9	17	1	EEPEPLSPELEYIPR
☑	45	1693.85	1692.85	1692.89	-0.04	1	6	31	1	VEDLHVGATVAPSSRR
☑	77	1968.02	1967.01	1967.03	-0.02	1	5	25	1	MAVARGVGSPEPAPPQLYK
☑	1	410.02	409.01							
☑	2	423.06	422.06							
☑	3	445.04	444.04							
☑	4	500.68	499.67							
☑	5	508.29	507.29							
☑	6	515.33	514.32							
☑	7	523.26	522.26							
☑	8	537.31	536.31							
☑	9	656.06	655.05							
☑	10	677.44	676.43							
☑	11	684.22	683.21							
☑	12	692.39	691.39							
☑	14	763.41	762.41							
☑	16	842.51	841.50							
☑	18	852.43	851.42							
☑	19	856.52	855.51							

Supplementary Fig. 1d. Mascot Search Results for B2 in Fig. 3c

Mascot Search Results

User : 14kDa
Email :
Search title :
MS data file : DATA.TXT
Database : NCBI_RefSeq_human RefSeq_human_170130 (111280 sequences; 74376932 residues)
Timestamp : 29 Oct 2020 at 08:25:32 GMT
Significant hits: gi|4557894 lysozyme C precursor [Homo sapiens]

Probability Based Mowse Score

Ions score is -10*Log(P), where P is the probability that the observed match is a random event.
Individual ions scores > 36 indicate identity or extensive homology (p<0.05).
Protein scores are derived from ions scores as a non-probabilistic basis for ranking protein hits.

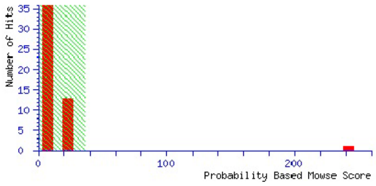

Peptide Summary Report

Switch to Protein Summary Report

To create a bookmark for this report, right click this link: Peptide Summary Report (../data/20201029/F062272.dat)

Select All | Select None | Search Selected | ☐ Error tolerant | Archive Report

1. gi|4557894 Mass: 16982 Score: 241 Peptides matched: 6
lysozyme C precursor [Homo sapiens]
☐ Check to include this hit in error tolerant search or archive report

	Query	Observed	Mr(expt)	Mr(calc)	Delta	Miss	Score	Expect	Rank	Peptide
☑	17	788.46	787.45	787.41	0.04	0	43	0.018	1	AWVAWR
☑	25	827.40	826.40	826.36	0.03	0	18	2.6	1	LGMDGYR + Oxidation (M)
☑	30	981.47	980.46	980.43	0.03	0	11	16	1	ATNYNAGDR
☑	31	1012.47	1011.46	1011.44	0.02	0	59	0.00017	1	WESGYNTR
☑	33	1039.63	1038.62	1038.59	0.03	1	7	18	1	VVRDPQGIR
☑	37	1400.70	1399.69	1399.67	0.02	0	103	8.6e-009	1	STDYGIFQINSR

Peptide matches not assigned to protein hits: (no details means no match)
List sorted by Decreasing Score. Sort by Decreasing Intensity or Increasing Query # / Mr

	Query	Observed	Mr(expt)	Mr(calc)	Delta	Miss	Score	Expect	Rank	Peptide
☑	20	804.45	803.44	803.41	0.03	0	23	1.9	1	QEVSSVR
☑	21	810.44	809.43	809.45	-0.02	1	17	3.8	1	REGPPVR
☑	11	678.45	677.44	677.40	0.04	1	13	2.9	1	ARYLR
☑	24	820.44	819.43	819.43	0.01	1	13	17	1	GMTKLDR
☑	23	814.47	813.46	813.43	0.03	0	10	25	1	APDLGVSR
☑	22	811.40	810.40	810.35	0.04	0	5	72	1	DDSVGYR
☑	14	685.42	684.41	684.42	-0.01	1	4	93	1	PELKAK
☑	1	445.21	444.21							
☑	2	464.43	463.42							
☑	3	514.34	513.33							
☑	4	530.32	529.32							
☑	5	589.77	588.77							
☑	6	606.32	605.31							
☑	7	631.35	630.35							
☑	8	634.34	633.33							
☑	9	656.12	655.12							
☑	10	662.39	661.38							
☑	12	680.85	679.84							
☑	13	684.28	683.27							
☑	15	742.45	741.45							
☑	16	744.48	743.47							
☑	18	789.46	788.45							
☑	19	802.44	801.43							
☑	26	832.42	831.41							
☑	27	833.43	832.43							
☑	28	836.45	835.44							
☑	29	842.54	841.53							
☑	32	1038.48	1037.47							
☑	34	1045.58	1044.57							
☑	35	1149.07	1148.06							

Supplementary Fig 1e. Mascot Search Results for B3 in Fig. 3c.

Mascot Search Results

User : YZ1
Email :
Search title :
MS data file : DATA.TXT
Database : NCBI_RefSeq_human RefSeq_human_170130 (111280 sequences; 74376932 residues)
Timestamp : 10 Dec 2020 at 04:23:56 GMT
Significant hits: gi|4758146 neutrophil defensin 1 preproprotein [Homo sapiens]
gi|4557894 lysozyme C precursor [Homo sapiens]

Probability Based Mowse Score

Ions score is -10*Log(P), where P is the probability that the observed match is a random event.
Individual ions scores > 35 indicate identity or extensive homology (p<0.05).
Protein scores are derived from ions scores as a non-probabilistic basis for ranking protein hits.

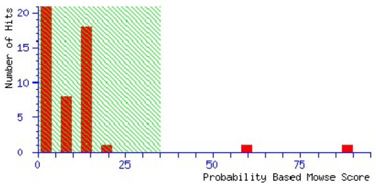

Peptide Summary Report

Switch to Protein Summary Report

To create a bookmark for this report, right click this link: Peptide Summary Report (../data/20201210/F062340.dat)

Select All | Select None | Search Selected | ☐ Error tolerant | Archive Report

1. gi|4758146 **Mass:** 10536 **Score:** 88 **Peptides matched:** 3
neutrophil defensin 1 preproprotein [Homo sapiens]
☐ Check to include this hit in error tolerant search or archive report

	Query	Observed	Mr(expt)	Mr(calc)	Delta	Miss	Score	Expect	Rank	Peptide
☑	21	986.51	985.50	985.50	0.00	0	40	0.02	1	IPACIAGER
☑	27	1117.52	1116.51	1116.50	0.01	0	28	0.22	1	YGTCIYQGR
☑	29	1273.61	1272.60	1272.60	-0.00	1	21	1.5	1	RYGTCIYQGR

Proteins matching the same set of peptides:
gi|4885179 **Mass:** 10580 **Score:** 88 **Peptides matched:** 3
neutrophil defensin 3 preproprotein [Homo sapiens]
gi|124248516 **Mass:** 10536 **Score:** 88 **Peptides matched:** 3
neutrophil defensin 1 isoform 2 preproprotein [Homo sapiens]
gi|695271700 **Mass:** 11323 **Score:** 88 **Peptides matched:** 3
neutrophil defensin 1 isoform 1 preproprotein [Homo sapiens]
gi|767950126 **Mass:** 11323 **Score:** 88 **Peptides matched:** 3
PREDICTED: neutrophil defensin 1 isoform X1 [Homo sapiens]
gi|767950128 **Mass:** 11367 **Score:** 88 **Peptides matched:** 3
PREDICTED: neutrophil defensin 3 isoform X1 [Homo sapiens]

---

2. gi|4557894 **Mass:** 16982 **Score:** 57 **Peptides matched:** 3
lysozyme C precursor [Homo sapiens]
☐ Check to include this hit in error tolerant search or archive report

	Query	Observed	Mr(expt)	Mr(calc)	Delta	Miss	Score	Expect	Rank	Peptide
☑	10	788.43	787.42	787.41	0.01	0	22	2.8	1	AWVAWR
☑	24	1012.44	1011.44	1011.44	-0.01	0	12	6.1	1	WESGYNTR
☑	30	1400.68	1399.67	1399.67	-0.00	0	23	0.66	1	STDYGIFQINSR

Peptide matches not assigned to protein hits: (no details means no match)
List sorted by Decreasing Score. Sort by Decreasing Intensity or Increasing Query # / Mr

	Query	Observed	Mr(expt)	Mr(calc)	Delta	Miss	Score	Expect	Rank	Peptide
☑	33	1502.70	1501.69	1501.68	0.00	1	11	8.1	1	MEEQQPEPKSQR + Oxidation (M)
☑	42	1765.76	1764.75	1764.79	-0.04	0	3	24	1	SAMGSSLYALESGSDFK + Oxidation (M)
☑	1	422.23	421.22							
☑	2	423.08	422.07							
☑	3	445.05	444.04							
☑	4	514.22	513.21							
☑	5	515.33	514.32							
☑	6	537.31	536.31							
☑	7	603.32	602.31							
☑	8	656.06	655.06							
	9	684.22	683.22							
