# Supplementary figures and images for: Cloaking the ACE2 receptor with salivary cationic proteins inhibits SARS-CoV-2 entry
Source: J Biochem. 2022 Jul 6;172(4):205–16. doi: 10.1093/jb/mvac054 (PMC9278198; doi:10.1093/jb/mvac054)

Supplementary Fig. 2

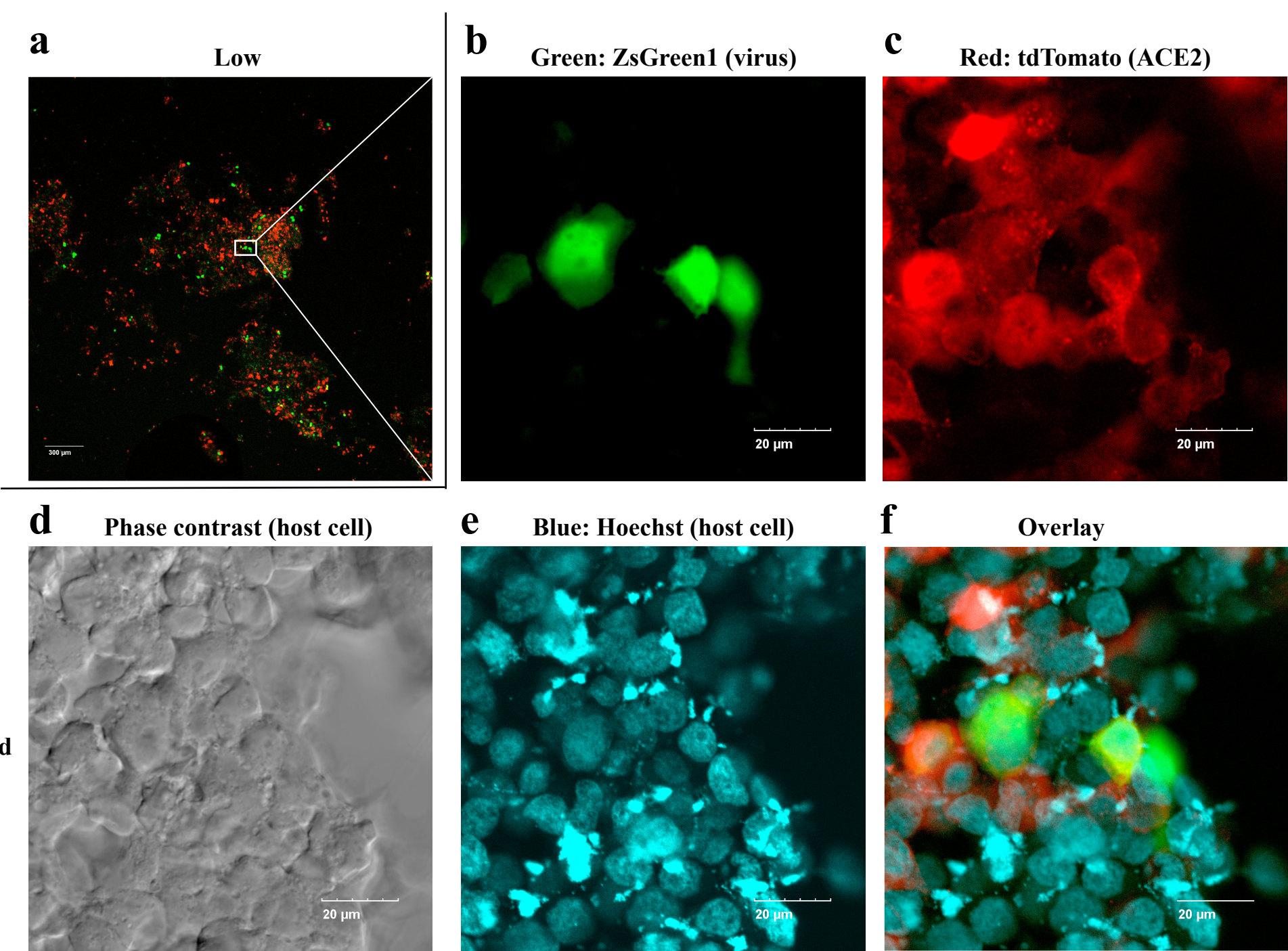

Supplement: Web_Material_mvac054 [file web_material_mvac054.zip › Supplementary Fig. 2 upload.pdf]
